# Supplementary material for: Silylium ion mediated 2+2 cycloaddition leads to 4+2 Diels-Alder reaction products
Source: Commun Chem. 2020 Sep 11;3:126. doi: 10.1038/s42004-020-00373-2 (PMC9814679; doi:10.1038/s42004-020-00373-2)
Supplement: Supplementary file 1 — Supplementary Information [file 42004_2020_373_MOESM1_ESM.pdf]

Silylium ion mediated 2+2 Cycloaddition leads to 4+2 Diels-Alder reaction products

Heng-Ding Wang, Hong-Jun Fan*.

State Key Laboratory of Molecular Reaction Dynamics, Dalian National Laboratory for Clean Energy, Dalian Institute of Chemical Physics, Chinese Academy of Sciences, Dalian 116023, China

University of Chinese Academy of Sciences, Beijing, China

Supplementary Note 1. Our proposed mechanism VS [4+2] mechanism

Recently Burcu Dedeoglu et al explored this mechanism with density functional theory (DFT), with their reduced model [they reduced more crowded triisopropylsilyl (TIPS) group into trimethylsilyl (TMS) group for simplicity]. They found that this reaction can proceed through stepwise mechanism[1]. Unfortunately, they chose **L-Ag⁺**, **sub1** and **sub2** as zero point directly, there was a coordinated complex **Ag-int1(TMS)** which is -5.96kcal/mol relative to zero point they used. We repeat their work with their reduced model and the barrier of stepwise mechanism is 25.4kcal/mol if use **L-Ag⁺**, **sub1** and **sub2** as reference point which is in accordance with their work. Also, if more energetically stable complex **Ag-int1(TMS)** use as the zero point, the barrier of stepwise mechanism is 31.3kcal/mol, which means this stepwise mechanism can even not proceeded in room temperature (Supplementary Fig. 1). Further, the stepwise mechanism fails to explain why electronic richer substrate **2c** and **2d** cannot give desired IEDDA product (Fig. 1b in the manuscript). Actually, they should be more active if this reaction can proceed through [4+2] stepwise mechanism.

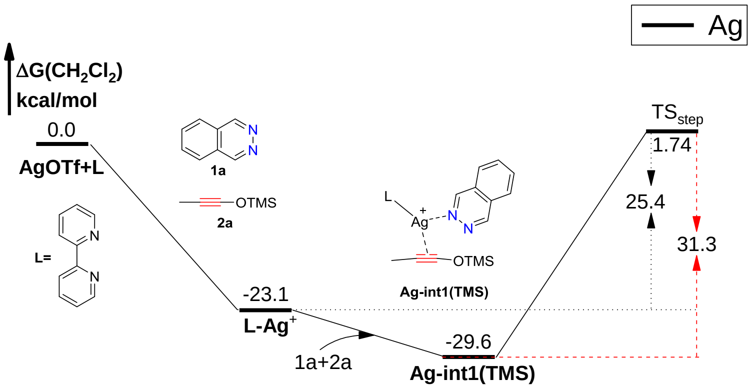

Supplementary Fig. 1. Reaction profiles of stepwise [4+2] cycloaddition mechanism of silver(I) catalyzed IEDDA with reduced model as ref 1. Gibbs energies are in kcal/mol.

To further verify that our proposed mechanism is much superior than stepwise mechanism, we repeated Burcu Dedeoglu's work [with sub2 reduced into trimethylchlorosilane (TMS)

substituted siloxy alkynes 2, under wB97XD/SDD,6-311+G(d,p)//M06-2X/SDD,6-31+G(d,p) level of theory]. We found that rate determine barrier of our proposed mechanism (**Ag-TS1**) lowed the stepwise mechanism (TSstep) 11.19kcal/mol which is a very strong evidence that our proposed mechanism is much more preferred. Further, we compared the rate determine barrier of our proposed mechanism with stepwise mechanism and found this advantage maintained under all the functional tested [2-5] (Supplementary Table 1).

Supplementary Table 1. Functional test of our proposed mechanism and stepwise mechanism use the same model as ref1.

functional	Mo62x	Wb97xd	Wb97xd	M06-d3	B3lyp-d3	B2plyp-d3	b2plypd3(bj)
Basis set	6-31+G(d,p)/ SDD	6-311+G(d,p)/ SDD	def2tzvp	def2tzvp	def2tzvp	def2tzvp	def2tzvp
TS_{step} /a.u.	-1659.8093	-1660.3360	-1660.5112	-1660.0226	-1661.0246	-1659.5461	-1659.5797
Ag-TS1/a.u.	-1659.8249	-1660.3535	-1660.5289	-1660.0395	-1661.0421	-1659.5625	-1659.5975
Δ G/kcal/mol	-10.30	-11.19	-9.76	-10.97	-11.10	-10.62	-10.95

The difference of concerted and stepwise mechanism is 7.2kcal/mol in the ref1, and only 0.7kcal/mol in our work. To make clear the cause of this difference, we compared reaction profile with the same model as ref1 (with TMS as protection group) under wb97xd/def2tzvp//M06-2x/def2tzvp,6-31G(d,p) level of theory with the same reference point as ref1. The reaction barrier obtained from wb97xd/def2tzvp//M06-2x/def2tzvp,6-31G(d,p) level of theory is in accordance with ref1 (the uncatalyzed barrier 46.6kcal/mol vs 45.2 in ref1, stepwise mechanism 27.0 vs 25.0 in ref1, concerted mechanism 32.9 vs 32.2). We further calculated reaction barrier with the same model under B2PLYPd3(BJ)/def2tzvp//M06-2x/def2tzvp,6-31G(d,p) level of theory and the barrier is in accordance with our work (uncatalyzed 41.6kcal/mol vs 40.0kcal/mol in our work, stepwise barrier 25.4kcal/mol vs 26.1kcal/mol in our work, concerted barrier 28.1kcal/mol vs 26.8kcal/mol). It turned out that energy differences of concerted and stepwise mechanism manly come from theoretical method to get the electronic energy (see details in Supplementary Table 2). For both uncatalyzed and catalyzed mechanism, the concerted barriers from wb97xd are higher than that from B2PLYPd3(BJ) by about 5kcal/mol, while the stepwise barriers are close. Since uncatalyzed mechanism is a pure organic system where double hybridized functional B2PLYPd3 is expected to work very well, we think although wb97xd is often quite suitable for metal catalyzed system, in this reaction the barrier of concerted mechanism may be overestimated. To avoid such functional induced errors, we further compared rate determine barrier of our proposed SMC mechanism and [4+2] mechanism with different functional (wb97xd, pbe0, b3lyp-d3, pbe0-d3, m06-d3, b2plyp, mpw2plyp, b2plyp-d3, b2plypd3(BJ), B3lyp see details in SI Supplementary Table 3) and found the rate determine barrier of SMC mechanism lowed [4+2] mechanism 9.2-16.2kcal/mol (with TIPS group as used in this work. The value will be 9.8-11.2kcal/mol, as shown in Supplementary Table 1, if TMS group is used as in ref1) among all the tested functional. Our proposed SMC mechanism is more favored than [4+2] mechanism for every functional that has been tested.

Supplementary Table 2. Gibbs free energy obtained with our model or the same model as ref1 under different level of theory with L-Ag^{+} sub1 and sub2 as reference point.

	Our work[a]	TMS[b]	TMS[c]	ref 1[d]
TS 0	40.0	41.6	46.6	45.2
TSstep	26.1	25.4	27.0	25.0
TScon	26.8	28.1	32.9	32.2

Gibbs energy obtained with L-Ag+, sub1, sub2 as reference point. The geometries optimized under m062x/6-31G(d,p),def2tzvp level of theory for a, b and c. (a). With triisopropylsilyl (TIPS) as protection group of sub2 and free energy correlated under b2plypd3/def2tzvp level of theory. (b). with Trimethylsilyl (TMS) group as protection group of sub2 and free energy correlated under b2plypd3/def2tzvp level of theory. (c). with Trimethylsilyl (TMS) group as protection group of sub2 and free energy correlated under wb97xd/def2tzvp level of theory. (d). ref1

Supplementary Table 3. Gibbs free energy correlated with different functional (with Ag-int1 as reference point and def2tzvp as basis set for all functional).

	PBE0	wb97xd	b3lyp-d3	pbe0-d3	m06-d3	b2plyp	mpw2plyp	b2plyp-d3	b2plypd3(BJ)	B3lyp
Ag-int1	0.0	0.0	0.0	0.0	0.0	0.0	0.0	0.0	0.0	0.0
Ag-TS1	23.6	21.3	20.1	19.9	22.5	23.2	23.8	20.5	19.5	25.7
Ag-TS2	16.4	18.9	20.2	13.2	21.6	20.7	21.4	18.4	16.9	25.1
TS0	48.7	44.4	44.2	39.6	43.1	48.2	48.0	40.8	40.0	58.1
TSstep	33.0	37.8	36.5	32.6	38.7	34.2	35.4	33.9	33.4	37.2

Supplementary method A. Comparation among M06, M06-d3, M06-2X optimized geometries.

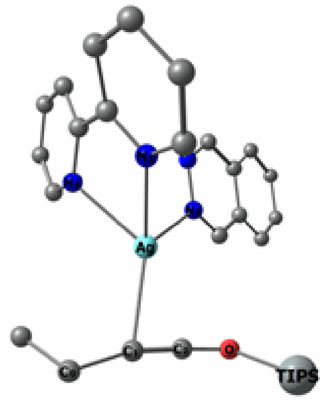

Ag-int1

Ag-int1	m06-2x	m06	m06-d3
C1-Ag	2.52	2.36	2.35
C2-Ag	2.59	2.504	2.49
N1-Ag	2.45	2.4	2.4
N2-Ag	2.40	2.4	2.4
N3-Ag	2.41	2.38	2.38
∠C0C1Ag	109.40	113.2	112.9
∠AgC2O	109.20	113.3	112.96
∠C1AgC2	27.50	29.06	29.22

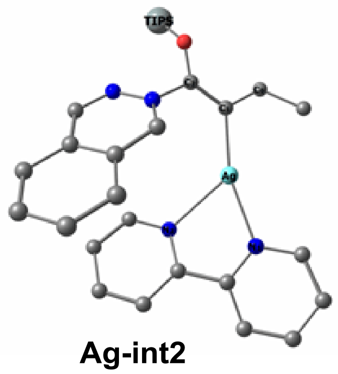

Ag-int2

Ag-int2	m06-2x	m06	m06-d3
C1-Ag	2.17	2.12	2.11
N1-Ag	2.39	2.36	2.27
N2-Ag	2.46	2.41	2.52
∠C0C1Ag	118.29	118.7	118.17
∠AgC1C2	126.26	124.73	124.39

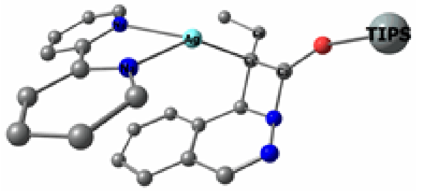

Ag-int3

Ag-int3	M06-2x	M06	M06-d3
C1-Ag	2.44	2.26	2.26
C2-Ag	2.8	2.79	2.77
N1-Ag	2.4	2.37	2.37
N2-Ag	2.4	2.35	2.35
∠C0C1Ag	113.4	116.1	115.9
∠AgC1C2	90.14	95.56	95.1

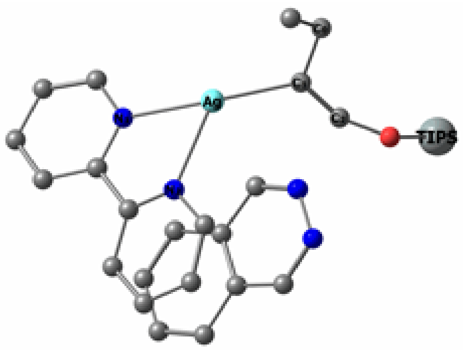

Ag-TS1

Ag-TS1	M06-2x	M06	M06-d3
C1-Ag	2.18	2.12	2.12
N1-Ag	2.34	2.27	2.28
N2-Ag	2.50	2.5	2.48
∠C0C1Ag	120.22	119.01	120.31
∠AgC1C2	121.66	124.46	122.49

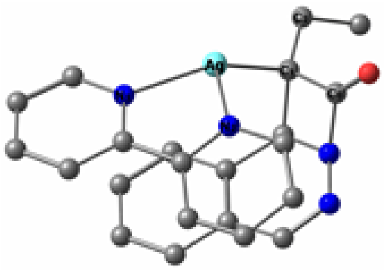

Ag-int4b

Ag-int4b	m06-2x	m06-d3
C1-Ag	2.25	2.18
N1-Ag	2.38	2.3
N2-Ag	2.51	2.52
∠C0C1Ag	96.62	98.48
∠AgC1C2	121.9	120.08

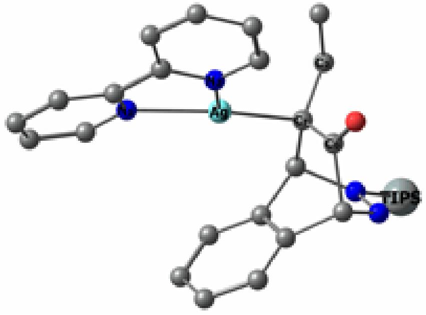

Ag-int5a

Ag-int5a	m06-2x	m06-d3
C1-Ag	2.3	2.22
N1-Ag	2.33	2.26
N2-Ag	2.45	2.44
∠C0C1Ag	98.88	95.36
∠AgC1C2	110.13	111.37

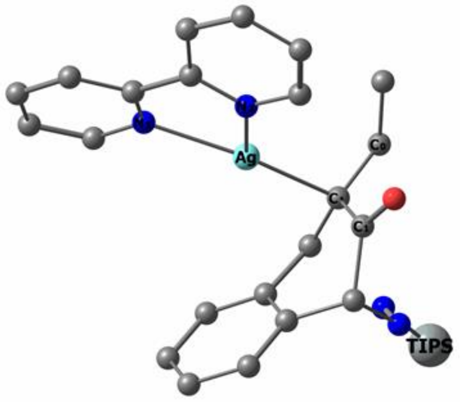

Ag-TS6a

Ag-TS6a	m06-2x	m06-d3
C1-Ag	2.38	2.23
C2-Ag	2.78	2.81
N1-Ag	2.33	2.26
N2-Ag	2.42	2.4
∠C0C1Ag	89.49	96.81
∠AgC1C2	108.69	110.3

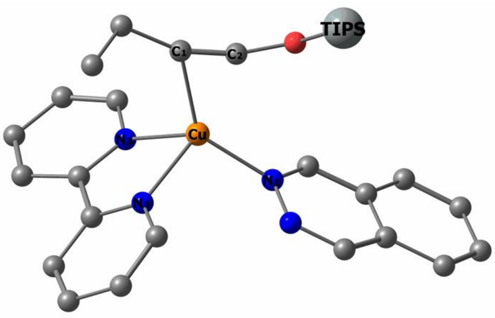

Cu-int1

Cu-int1	m06-2x	m06-d3
C1-Cu	2.07	1.97
C2-Cu	2.09	1.97
N1-Cu	2.03	1.95
N2-Cu	2.15	2.06
N3-Cu	2.14	2.14
∠C0C1Cu	34.46	36.9

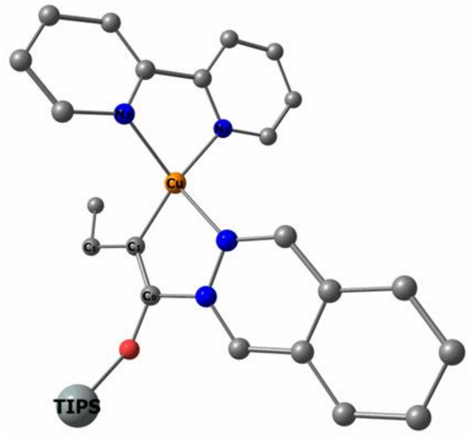

Cu-int2

Cu-int2	m06-2x	m06-d3
C1-Cu	1.96	1.91
N1-Cu	2.05	1.97
N2-Cu	2.07	1.99
∠C0C1Cu	115.34	112.18
∠CuC1C2	127.01	128.21

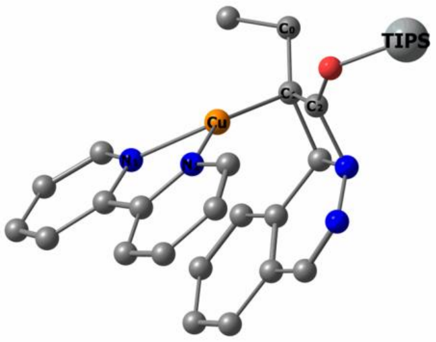

Cu-int3

Cu-int3	m06-2x	m06-d3
C1-Cu	2.02	1.95
N1-Cu	2.05	1.99
N2-Cu	2.01	1.96
∠C0C1Cu	120.06	122.24
∠CuC1C2	73.68	71.51

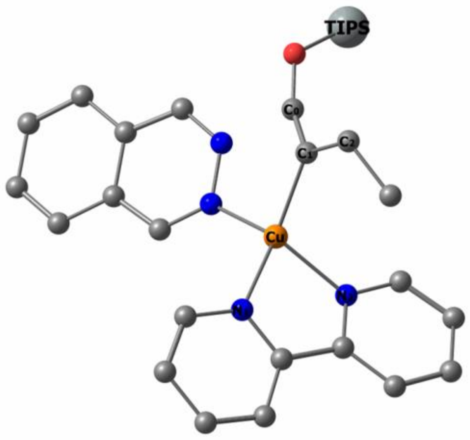

Cu-TS1

Cu-TS1	m06-2x	m06-d3
C1-Cu	2.03	1.99
N0-Cu	2.09	1.97
N1-Cu	2.08	2
N2-Cu	2.07	2
∠C0C1Cu	115.44	115.99
∠CuC1C2	120.06	122.1

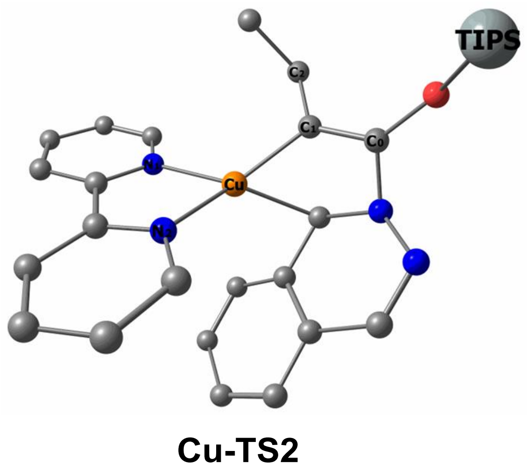

Cu-TS2	m06-2x	m06-d3
C1-Cu	1.95	1.92
N1-Cu	2.10	2.04
N2-Cu	2.10	2.04
∠C0C1Cu	116.92	119.56
∠CuC1C2	117.91	118.03

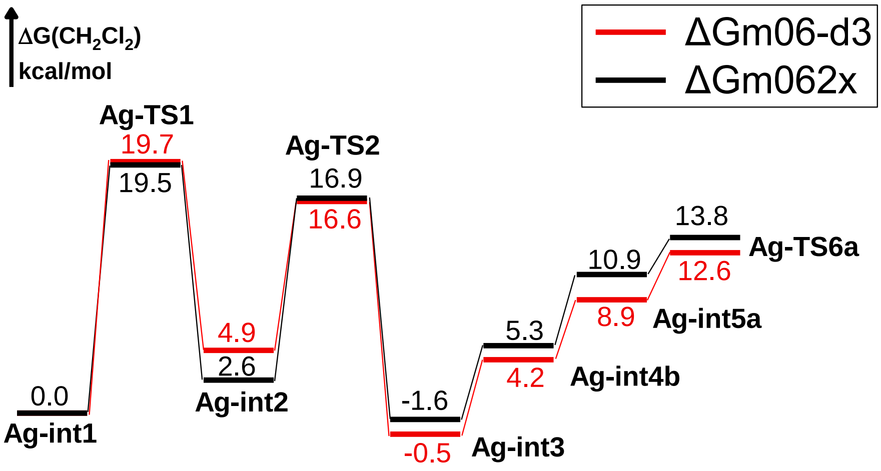

Supplementary Fig. 2. Reaction profiles of SMC mechanism [Silver(I) catalyzed]. Black line, B2PLYPd3(BJ)/def2tzvp//M06-2X/def2tzxp,6-31G(d,p) level of theory. Red line B2PLYPd3(BJ)/def2tzvp//M06-d3/def2tzxp,6-31G(d,p) level of theory.

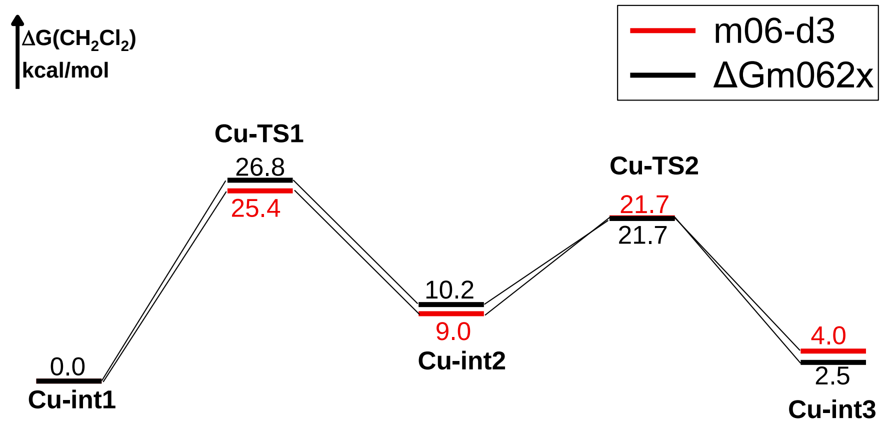

Supplementary Fig. 3. Reaction profiles of SMC mechanism [Copper(I) catalyzed]. Black line, B2PLYPd3(BJ)/def2tzvp//M06-2X/6-31G(d,p) level of theory. Red line B2PLYPd3(BJ)/def2tzvp//M06-d3/ 6-31G(d,p) level of theory.

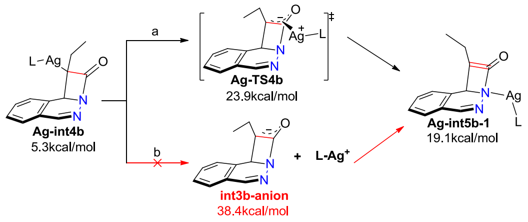

Supplementary Fig. 4. Isomerization of **Ag-int4b** (ketone form) into **Ag-int5b-1**(enol form). Gibbs free energies are in kcal/mol.

Supplementary method B. IRC calculation of key transition states.

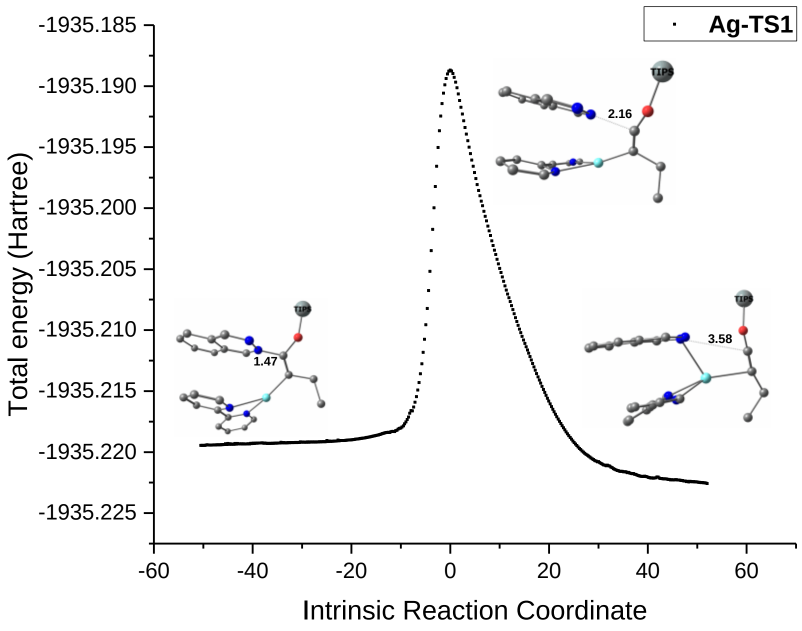

Supplementary Fig. 5. IRC calculation of Ag-TS1.

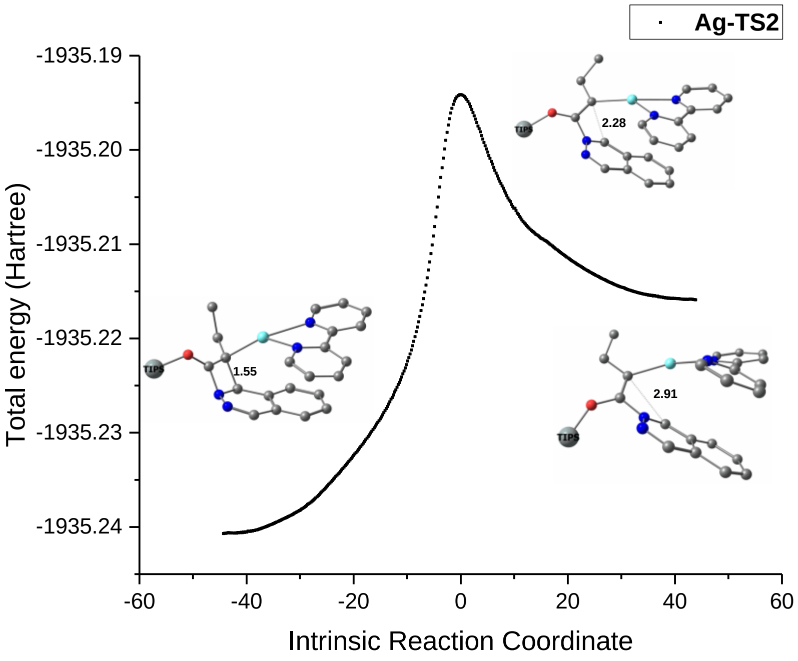

Supplementary Fig. 6. IRC calculation of Ag-TS2.

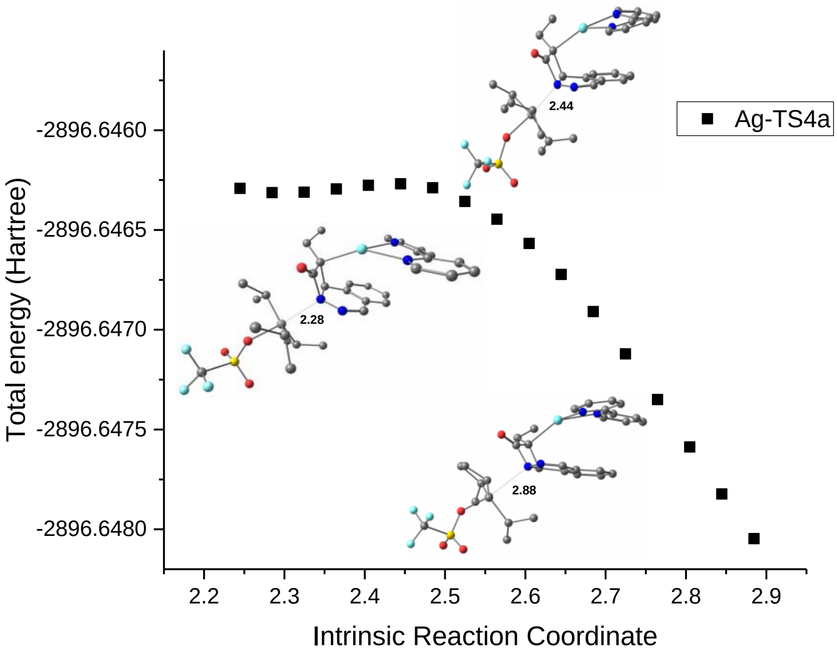

Supplementary Fig. 7. IRC calculation of Ag-TS4a. There is a five coordinated intermediate after Ag-TS4a, the energy of which is only 0.028kcal/mol lower than Ag-TS4a. The dissociation of this complex lead to Ag-int4b and TIPS-OTf is entropy driving process. The Ag-TS3 can be obtained

directly through QST3 method, but the IRC calculation failed perhaps due to the extremely low imaginary frequency (-41.5cm-1). Therefore, we scanned coordinate of Ag-TS3 through the reaction coordinate.

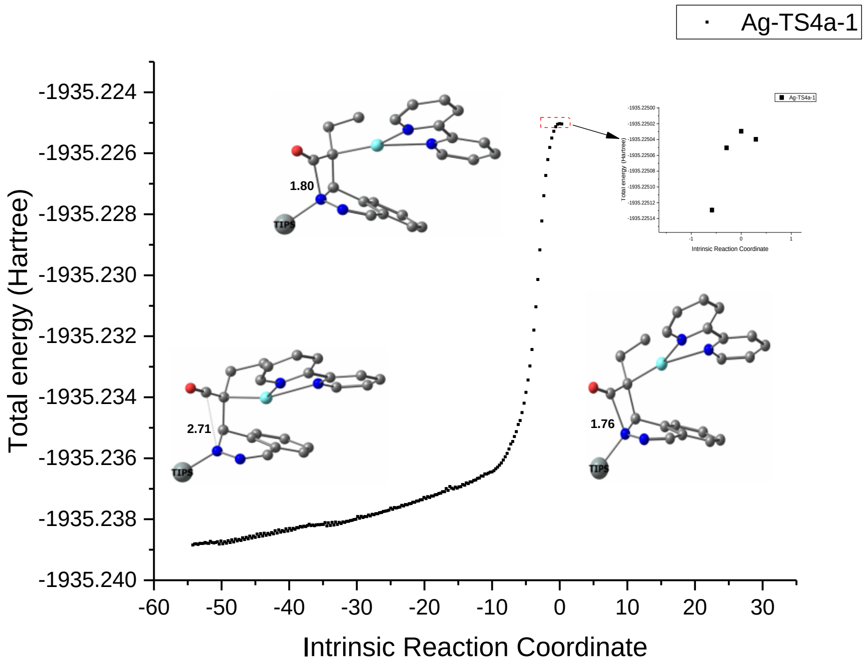

Supplementary Fig. 8. IRC calculation of Ag-TS4a-1， note that this transition state is very close to its reactant.

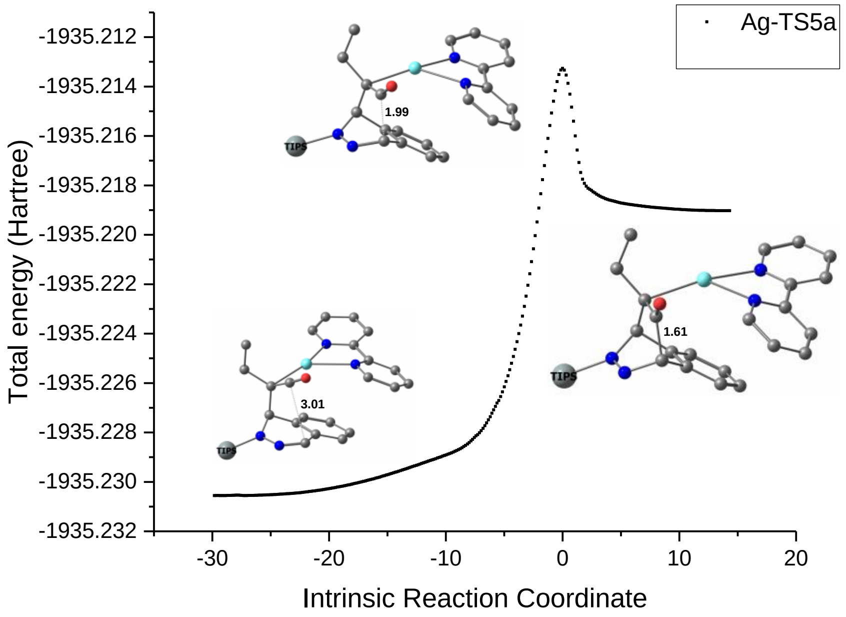

Supplementary Fig. 9. IRC calculation of Ag-TS5a

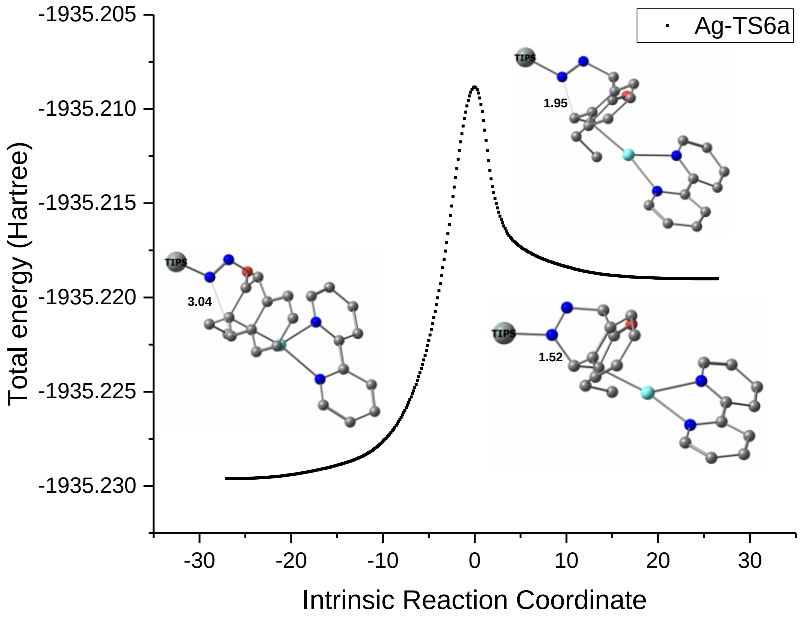

Supplementary Fig. 10. IRC calculation of Ag-TS6a

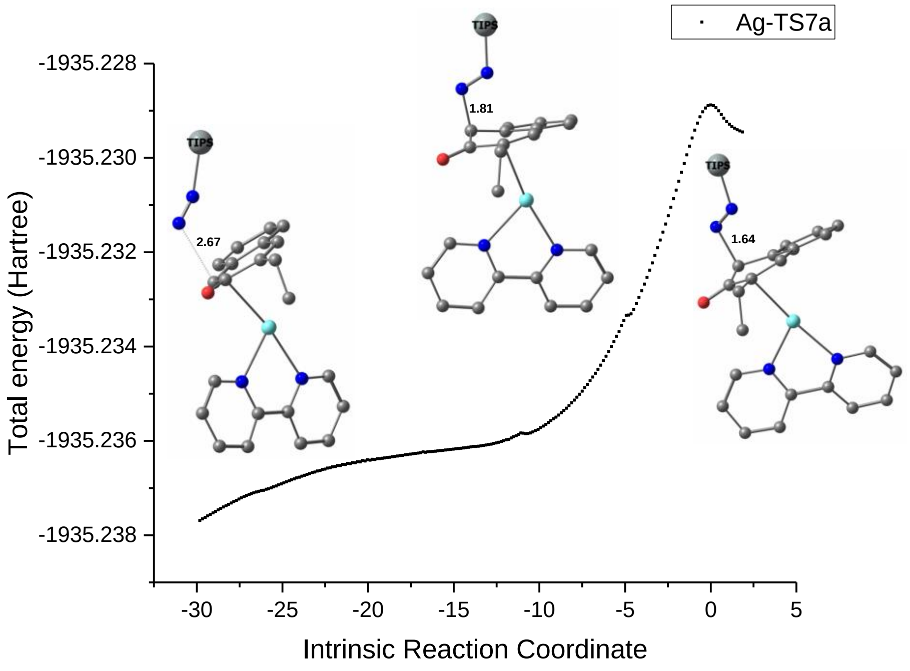

Supplementary Fig. 11. IRC calculation of Ag-TS7a

Supplementary Referance

1. Avcı, Ö. N.; Catak, S.; Dereli, B.; Aviyente, V.; Dedeoglu, B., Elucidation of the Mechanism of Silver-Catalyzed Inverse Electron-Demand Diels-Alder (IEDDA) Reaction of 1,2-Diazines and Siloxy Alkynes. *ChemCatChem*. **12**, 366–372 (2020).

2. Weigend, F.; Ahlrichs, R., Balanced basis sets of split valence, triple zeta valence and quadruple zeta valence quality for H to Rn: Design and assessment of accuracy. *Physical Chemistry Chemical Physics* **2005,** *7* (18), 3297-3305.
3. Grimme, S.; Antony, J.; Ehrlich, S.; Krieg, H., A consistent and accurate ab initio parametrization of density functional dispersion correction (DFT-D) for the 94 elements H-Pu. *The Journal of chemical physics* **2010,** *132* (15), 154104.
4. Zhao, Y.; Truhlar, D. G., The M06 suite of density functionals for main group thermochemistry, thermochemical kinetics, noncovalent interactions, excited states, and transition elements: two new functionals and systematic testing of four M06-class functionals and 12 other functionals. *Theoretical Chemistry Accounts* **2008,** *120* (1), 215-241.
5. Binkley, J. S.; Pople, J. A.; Hehre, W. J., Self-consistent molecular orbital methods. 21. Small split-valence basis sets for first-row elements. *Journal of the American Chemical Society* **1980,** *102* (3), 939-947.
