# Supplementary material for: Silylium ion mediated 2+2 cycloaddition leads to 4+2 Diels-Alder reaction products
Source: Commun Chem. 2020 Sep 11;3:126. doi: 10.1038/s42004-020-00373-2 (PMC9814679; doi:10.1038/s42004-020-00373-2)
Supplement: Supplementary file 2 — Description of Additional Supplementary Files [file 42004_2020_373_MOESM2_ESM.pdf]

Description of Additional Supplementary Files

File Name: Supplementary Data1
Description: Cartesian coordinates file for compound sub1

File Name: Supplementary Data2
Description: Cartesian coordinates file for compound sub2

File Name: Supplementary Data3
Description: Cartesian coordinates file for compound L

File Name: Supplementary Data4
Description: Cartesian coordinates file for compound L-Ag

File Name: Supplementary Data5
Description: Cartesian coordinates file for compound L-Cu

File Name: Supplementary Data6
Description: Cartesian coordinates file for compound CuOTf

File Name: Supplementary Data7
Description: Cartesian coordinates file for compound AgOTf

File Name: Supplementary Data8
Description: Cartesian coordinates file for compound Ag-int1

File Name: Supplementary Data9-
Description: Cartesian coordinates file for compound Ag-int2

File Name: Supplementary Data10
Description: Cartesian coordinates file for compound Ag-int3

File Name: Supplementary Data11
Description: Cartesian coordinates file for compound Ag-TS1

File Name: Supplementary Data12
Description: Cartesian coordinates file for compound Ag-TS2

File Name: Supplementary Data13
Description: Cartesian coordinates file for compound Ag-TS3

File Name: Supplementary Data14
Description: Cartesian coordinates file for compound Ag-int4a

File Name: Supplementary Data15
Description: Cartesian coordinates file for compound Ag-int4a-1

File Name: Supplementary Data16
Description: Cartesian coordinates file for compound Ag-int5a

File Name: Supplementary Data17
Description: Cartesian coordinates file for compound Ag-int6a

File Name: Supplementary Data18
Description: Cartesian coordinates file for compound Ag-int7

File Name: Supplementary Data19
Description: Cartesian coordinates file for compound Ag-TS3

File Name: Supplementary Data20
Description: Cartesian coordinates file for compound Ag-TS3a

File Name: Supplementary Data21
Description: Cartesian coordinates file for compound Ag-TS4a

File Name: Supplementary Data22
Description: Cartesian coordinates file for compound Ag-TS4a-1

File Name: Supplementary Data23
Description: Cartesian coordinates file for compound Ag-TS5a

File Name: Supplementary Data24
Description: Cartesian coordinates file for compound Ag-TS6a

File Name: Supplementary Data25
Description: Cartesian coordinates file for compound Ag-TS7a

File Name: Supplementary Data26
Description: Cartesian coordinates file for compound Ag-intb

File Name: Supplementary Data27
Description: Cartesian coordinates file for compound Ag-int5b-1

File Name: Supplementary Data28
Description: Cartesian coordinates file for compound Ag-int5b-2

File Name: Supplementary Data29
Description: Cartesian coordinates file for compound Ag-int6b

File Name: Supplementary Data30
Description: Cartesian coordinates file for compound Ag-TS4b

File Name: Supplementary Data31
Description: Cartesian coordinates file for compound Ag-TS5b

File Name: Supplementary Data32
Description: Cartesian coordinates file for compound Ag-TS6b

File Name: Supplementary Data33
Description: Cartesian coordinates file for compound Ag-TS7b

File Name: Supplementary Data34
Description: Cartesian coordinates file for compound Cu-int1

File Name: Supplementary Data35
Description: Cartesian coordinates file for compound Cu-int2

File Name: Supplementary Data36
Description: Cartesian coordinates file for compound Cu-int3

File Name: Supplementary Data37
Description: Cartesian coordinates file for compound Cu-TS1

File Name: Supplementary Data38
Description: Cartesian coordinates file for compound Cu-TS2

File Name: Supplementary Data39
Description: Cartesian coordinates file for compound Cu-int4a-1

File Name: Supplementary Data40
Description: Cartesian coordinates file for compound Cu-int5a

File Name: Supplementary Data41
Description: Cartesian coordinates file for compound Cu-TS3a

File Name: Supplementary Data42
Description: Cartesian coordinates file for compound Cu-TS4a

File Name: Supplementary Data43
Description: Cartesian coordinates file for compound Cu-TS5a

File Name: Supplementary Data44
Description: Cartesian coordinates file for compound Cu-int4b

File Name: Supplementary Data45
Description: Cartesian coordinates file for compound Cu-int5b-1

File Name: Supplementary Data46
Description: Cartesian coordinates file for compound Cu-int5b-2

File Name: Supplementary Data47

Description: Cartesian coordinates file for compound Cu-int6b

File Name: Supplementary Data48
Description: Cartesian coordinates file for compound Cu-TS4b

File Name: Supplementary Data49
Description: Cartesian coordinates file for compound Cu-TS6b

File Name: Supplementary Data50
Description: Cartesian coordinates file for compound Cu-TS7b

File Name: Supplementary Data51
Description: Cartesian coordinates file for compound Ag-TScon

File Name: Supplementary Data52
Description: Cartesian coordinates file for compound Ag-TSstep

File Name: Supplementary Data53
Description: Cartesian coordinates file for compound Cu-TScon

File Name: Supplementary Data54
Description: Cartesian coordinates file for compound Cu-TSstep

File Name: Supplementary Data55
Description: Cartesian coordinates file for compound 4m-int1

File Name: Supplementary Data56
Description: Cartesian coordinates file for compound 4m-TS1

File Name: Supplementary Data57
Description: Cartesian coordinates file for compound 8m-int2

File Name: Supplementary Data58
Description: Cartesian coordinates file for compound 8m-TS2

File Name: Supplementary Data59
Description: Cartesian coordinates file for compound 1,2-NCOT

File Name: Supplementary Data60
Description: Cartesian coordinates file for compound 8m-TS3

File Name: Supplementary Data61
Description: Cartesian coordinates file for compound 8m-TS3-1

File Name: Supplementary Data62
Description: Cartesian coordinates file for compound BCB

File Name: Supplementary Data63
Description: Cartesian coordinates file for compound NSBV
